# Timing of Epidural Placement After an Epidural Blood Patch

**DOI:** 10.1155/cria/1692478

**Published:** 2025-06-09

**Authors:** Tara L. Kelly, William Barrett, Amanda Bunnell, Toby B. Steinberg, Christopher Wolla

**Affiliations:** Department of Anesthesiology and Perioperative Medicine, Medical University of South Carolina, Charleston, South Carolina, USA

## Abstract

Epidural placement is a mainstay in the pain management of patients undergoing brachytherapy treatment of cervical cancer. A significant number of patients undergo multiple treatment cycles over the course of several weeks, necessitating repeated neuraxial catheter placements. This increases the risk of complications, including postdural puncture headaches, which may require further neuraxial instrumentation for the administration of an epidural blood patch. We present the case of a patient who received an epidural blood patch for postdural puncture headache in the middle of her brachytherapy treatment series. There is limited literature addressing the optimal timing for subsequent epidural catheter placement after an epidural blood patch.

## 1. Introduction

In 2020, cervical cancer was both the fourth most diagnosed and fourth leading cause of cancer-related death in women worldwide [1]. One possible treatment for cervical cancer involves the placement of an interstitial or intracavitary radiation source for brachytherapy treatments [2]. Patients undergoing this form of care often require multiple sessions of brachytherapy over an extended period, necessitating repeated administrations of anesthesia for radiation applicator placement and analgesia throughout the course of treatment [2, 3]. One of the preferred ways to provide anesthesia and analgesia in these patients is with epidural catheter placement [3]. As with any procedure, there are possible complications with epidural catheter placement such as bleeding, infection, damage to neural structures, and postdural puncture headache (PDPH) [4].

Epidural blood patch (EBP) is the gold standard treatment for PDPH, particularly for patients who have failed conservative management [5]. The mechanism of action is thought to be physical compression of the thecal space leading to a rise in cerebral spinal fluid (CSF) pressure, thereby alleviating the headache. This is thought to be followed by clot formation which closes the dural puncture eliminating the CSF leak with a success rate of 70%–98% [5]. There has been significant research on the indications for EBP, the technique, and possible complications [6–8], but there is a lack of information on the timing and placement of epidural after EBP.

For patients requiring frequent epidural placement for procedures such as brachytherapy, there is an inherently increased risk for complications including PDPH, potentially necessitating treatment with EBP. However, little guidance exists for questions surrounding the timing and efficacy of subsequent epidural placement for procedures required shortly after EBPs. Furthermore, there is limited guidance available regarding the management of these patients when considering subsequent epidural placements. We present the case of a patient requiring epidural placement for analgesia for tandem and ovoid brachytherapy after a recent EBP for PDPH.

## 2. Case Presentation

A 46-year-old female with normal body habitus and cervical cancer presented for placement of tandem and ovoid for brachytherapy, for which an epidural catheter was placed for analgesia for radiation treatments. The initial epidural catheter was placed prior to the procedure in the preoperative holding area. Placement was difficult requiring 3 attempts and with the initial 2 attempts at L4-5 interspace, and subsequent successful placement of the epidural catheter at L3-4 interspace. There was no reported CSF return on any of the attempts. The patient reported successful analgesia, and the catheter was then removed on Catheter day 1 without complication. The patient was then discharged by the primary team.

Six days later, the patient returned for her second round of radiation therapy. At this time, she reported that she had experienced an initially minor headache that had escalated to a severe postural headache with nausea, blurred vision, dizziness, light sensitivity, and hearing loss. She was evaluated by the anesthesia team, who determined that she likely had a PDPH, despite no known dural puncture. After a discussion with the patient and the gynecology–oncology team, the decision was made to perform an EBP. The EBP was performed with ease at the L3-4 level with 20 ccs total blood volume. This resulted in the resolution of the patient's symptoms.

Though our patient's symptoms were significantly improved after her EBP, she still required the next round of radiation therapy. She presented to the preoperative holding area 8 days after her EBP. The epidural placement was again difficult, requiring 5 total attempts, by our regional anesthesia fellow and then a senior regionally trained anesthesia attending. On the first attempt, there was a suspected inadvertent dural puncture at the L3-4 level with a small amount of CSF return. The second attempt at the L2-3 level was complicated by positive aspiration of heme after catheter placement. The third attempt at the L2-3 level had negative aspiration of heme after catheter placement but there was a positive test dose with increased HR by 15 beats per minute and the patient reported tinnitus. The fourth attempt at the L4-5 level was complicated by positive heme aspiration after catheter placement. The fifth attempt was finally successful with negative aspiration for heme and CSF and a negative test dose. The epidural provided appropriate analgesia for the duration of radiation therapy. The epidural was removed on Catheter day 1 and there were no postprocedural complications despite complicated placement. The patient was discharged and did not require additional brachytherapy treatments.

## 3. Discussion

Epidural catheter placement for tandem and ovoid brachytherapy is a mainstay for patients to achieve adequate analgesia, limit narcotics, and avoid excessive nausea for the duration of the radiation treatments [3]. Patients undergoing this type of regimen will require frequent epidural placements, thereby increasing the cumulative risk of dural puncture and subsequent PDPH, which may necessitate an EBP. As in our case, these patients who received an EBP may present for further brachytherapy treatment requiring epidural catheter placement.

A review of the literature offers limited guidance on the optimal timing for epidural catheter placement to provide analgesia following EBP. One review article discusses epidural steroid injection for chronic pain, noting that placing an epidural after EBP risks infection, epidural placement failure, and disruption of the EBP [9]. These risks are crucial to consider when determining the optimal timing between epidural procedures; however, this review did not address the timing of these interventions. One study examining the scar formation and healing process on the dura of Angora goats following dural puncture and subsequent blood patch demonstrated that scar tissue is maximally thick at 2 weeks postblood patch placement and dura returns to normal thickness at about 3 months [10]. Another case report showed successful epidural placement in a laboring patient 3 days after her EBP. In this case, a 29-year-old female underwent combined spinal–epidural (CSE) for labor analgesia placed 3 days after her EBP. The CSE was successfully placed and provided adequate analgesia for the duration of her labor [11]. Not every case has achieved successful analgesia with epidurals placed after EBP. A laboring patient with EBP placed 3 years prior only received limited epidural coverage. The new epidural in this case only provided analgesia up to L2 dermatome [12].

In our case, we consulted with the gynecology–oncology team to determine the longest permissible interval between the EBP and subsequent epidural catheter placement. This led to delaying her second round of radiation therapy. Given the importance of timing in the treatment plan for cervical cancer, we agreed upon an 8-day interval. Our patient received adequate analgesic coverage throughout her radiation treatments. She proved to be a challenging case for epidural catheter placement during both the initial and second treatment cycles; however, she experienced no symptoms of PDPH following her final treatment. We speculate that our second epidural catheter placement could have been increasingly difficult because of the alteration of the epidural space from the prior EBP placement. We do not have imaging to prove this theory, but we had experienced anesthesiologists performing the procedure, and had aspiration of CSF, heme, and positive test doses. While the optimal timing for epidural catheter placement after an EBP remains uncertain, we had success with an 8-day interval.

Given the limited available information, the dura takes about 3 months to return to its normal level of thickness. It is unclear whether it is necessary to wait for that duration, as there are cases, including our own, demonstrating successful epidural analgesia after EBP with a significantly shorter period between procedures. On the other hand, EBP could alter the epidural space, limiting the spread of future epidural coverage. With the increasing use of repeated epidural placements for scheduled radiation treatments, further research is needed to investigate the optimal interval between EBP and subsequent epidural catheter placement.

## Data Availability

Data sharing is not applicable to this article as no datasets were generated or analyzed during the current study.

## Nomenclature

PDPH: Postdural puncture headache
EBP: Epidural blood patch
CSE: Combined spinal–epidural
CSF: Cerebral spinal fluid
